# HIV-1 infection alters CD4^+^ memory T-cell phenotype at the site of disease in extrapulmonary tuberculosis

**DOI:** 10.1002/eji.201141927

**Published:** 2011-10-05

**Authors:** Kerryn Matthews, Mpiko Ntsekhe, Faisal Syed, Thomas Scriba, James Russell, Kemi Tibazarwa, Armin Deffur, Willem Hanekom, Bongani M Mayosi, Robert J Wilkinson, Katalin A Wilkinson

**Affiliations:** 1 Clinical Infectious Diseases Research Initiative, Institute of Infectious Diseases and Molecular Medicine, University of Cape TownObservatory, South Africa; 2 Department of Medicine, Faculty of Health Sciences, University of Cape TownObservatory, South Africa; 3 South African Tuberculosis Vaccine Initiative and School of Child and Adolescent Health, Institute of Infectious Diseases and Molecular Medicine, University of Cape TownObservatory, South Africa; 4 MRC National Institute for Medical ResearchLondon, UK; 5 Department of Medicine, Imperial College LondonUK

**Keywords:** CD4^+^ T cells, HIV-1, *Mycobacterium tuberculosis*, Pericarditis

## Abstract

HIV-1-infected people have an increased risk of developing extrapulmonary tuberculosis (TB), the immunopathogenesis of which is poorly understood. Here, we conducted a detailed immunological analysis of human pericardial TB, to determine the effect of HIV-1 co-infection on the phenotype of *Mycobacterium tuberculosis* (MTB)-specific memory T cells and the role of polyfunctional T cells at the disease site, using cells from pericardial fluid and blood of 74 patients with (*n*=50) and without (*n*=24) HIV-1 co-infection. The MTB antigen-induced IFN-γ response was elevated at the disease site, irrespective of HIV-1 status or antigenic stimulant. However, the IFN-γ ELISpot showed no clear evidence of increased numbers of antigen-specific cells at the disease site except for ESAT-6 in HIV-1 uninfected individuals (*p*=0.009). Flow cytometric analysis showed that CD4^+^ memory T cells in the pericardial fluid of HIV-1-infected patients were of a less differentiated phenotype, with the presence of polyfunctional CD4^+^ T cells expressing TNF, IL-2 and IFN-γ. These results indicate that HIV-1 infection results in altered phenotype and function of MTB-specific CD4^+^ T cells at the disease site, which may contribute to the increased risk of developing TB at all stages of HIV-1 infection.

## Introduction

Persons with human immunodeficiency virus (HIV-1) infection are at an increased risk of developing tuberculosis (TB), especially extrapulmonary TB 1, 2. It is important to understand the development of extrapulmonary TB, as its pathogenesis differs from pulmonary disease 3, 4. Little is known about lymphocyte recruitment, the immunological response, or the effect of HIV-1 infection on the immunopathogenesis at disease sites of extrapulmonary TB.

*Mycobacterium tuberculosis* (MTB) infection of the pericardium results in accumulation of fluid around the heart, or fibrosis of the pericardium, with a high mortality rate 5. Pericardial effusions are generally lymphocytic with CD4^+^ T cells dominating in HIV-1 uninfected and CD8^+^ T cells in HIV-1-infected pericardial TB patients 6. However, the detailed phenotype of T cells at the disease site has never been investigated.

Distinct memory T-cell populations in humans can be phenotypically characterized using the combination of markers such as CCR7, CD62L, CD45RA, CD27 and CD28 7–10. We have previously shown that the antigen-specific T cells at the disease site in pleural TB are predominantly CD4^+^, able to exert effector function rapidly (by releasing IFN-γ within 6 h after antigen contact), and lack the chemokine receptor CCR7 11. It is not known, however, how HIV-1 co-infection affects the phenotype of MTB-specific memory T cells at the disease site. It has been shown that HIV-1 infection can affect the phenotype of CD4^+^ T cells specific for cytomegalovirus (CMV) in the blood of persons co-infected with HIV-1 and CMV towards a less differentiated state 12. This finding suggests that HIV-1 infection affects the phenotype of CD4^+^ T cells specific for other pathogens, resulting in reduced ability of the immune system to control other, co-infecting pathogens, and thereby opportunistic infections.

CD4^+^ T cells secreting IFN-γ play an essential role in protective immunity against TB 13. However, the recent evidence suggests that polyfunctional CD4^+^ T cells secreting IFN-γ in combination with other cytokines, such as tumor necrosis factor (TNF) and interleukin (IL)-2, may also contribute 14–25. These cells can be found in the blood of HIV-1-infected people, but their ability to secrete more than one cytokine decreases with increasing HIV-1 viral load 26. The polyfunctionality of blood CD4^+^ T cells specific for MTB is restored by antiretroviral treatment 27. HIV-1 infection severely impairs the frequency of polyfunctional cells in the bronchoalveolar lavage of people with latent TB 28, but whether these T cells are present at TB disease sites, or what effect HIV-1 co-infection has, is not known.

Here, we describe the effect of HIV-1 co-infection on extrapulmonary TB in patients with pericardial TB. We specifically determined the effect of HIV-1 on the phenotype of MTB-specific memory cells at the disease site, as well as the role of polyfunctional T cells at the disease site. We found that HIV-1 infection results in altered phenotype and function of MTB-specific CD4^+^ T cells at the site of disease towards a less differentiated and more polyfunctional phenotype. These differences may relate to the increased susceptibility to TB at all stages of HIV-infection.

## Results

### Characterization of pericardial TB patients at baseline

A total of 24 HIV-1-uninfected and 50 HIV-1-infected patients with probable or definite pericardial TB were included in this study. The baseline characteristics of the patients are summarized in Table 1. HIV-1-infected patients presented with TB pericarditis at a much younger age (median: 31; range: 20–66), compared with the HIV-1-uninfected patients (median: 54: range: 19–80: *p*<0.0001). There was no difference in gender and TB diagnosis classification (definite/probable) between HIV-1-uninfected and infected patients. In the HIV-1-infected patients with available CD4^+^ counts (*n*=38), the median was 109 (IQR 76–278) cells/μL. CD4^+^ count information was also available for 18 HIV-uninfected patients, with a median of 439 (IQR: 340–649), significantly higher than the HIV-infected group (*p*<0.0001). One HIV-1-infected patient was taking antiretroviral treatment of two weeksŉ duration at the time of diagnosis.

**Table 1:** Characteristics of the patients included in the study

	HIV-1 uninfected	HIV-1 infected	*p*-Valuea)
Number	24	50	
Age (range) years	54 (19–80)	31 (20–66)	*p*<0.0001
Gender: Male/Female	18/6	28/22	*p*=0.13
TB diagnosis: definite/probable	14/10	26/24	*p*=0.63
CD4 count (IQR)	439 (340–649) [*n*=18]	109 (76–278) [*n*=38]	*p*<0.0001b)
Number on anti retroviral treatment	n/a	1	N/a

a) Indicating differences between the HIV-1-uninfected and infected groups of patients, using Fisherŉs exact test.

b) CD4 counts between the HIV-1-uninfected and infected groups were compared using the Mann–Whitney *U* test.

### Increased IFN-γ secretion in pericardial fluid compared with blood, irrespective of HIV-1 status

**Figure 1:**
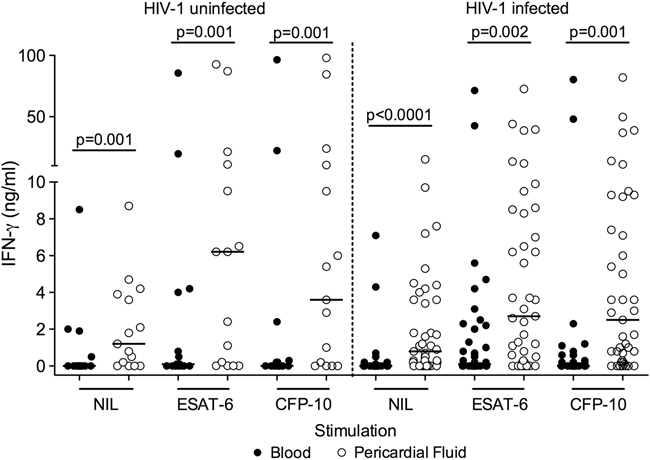
Pericardial TB patients have increased antigen-specific IFN-γ secretion in the pericardial fluid compared with blood. Undiluted blood and pericardial fluid were cultured overnight either unstimulated (NIL) or stimulated with ESAT-6 and CFP-10. IFN-γ concentration (ng/mL) was determined by ELISA and bars represent medians. Each circle is representative of one sample. *P*-Values indicate the differences between blood and pericardial fluid using the Wilcoxon-matched pairs test.

First, we compared the IFN-γ secretion of whole blood and pericardial fluid stimulated overnight with MTB-specific antigens in 15 HIV-1-uninfected and 41 HIV-1-infected patients (Fig. 1). The median concentration of IFN-γ was significantly higher in the unstimulated pericardial fluid compared with that in blood in both groups of patients (1.2 ng/mL IQR 0–3.9 and 0.8 ng/mL IQR 0.1–3.6 in HIV-1-uninfected and infected pericardial fluid respectively, both *p*≤0.001 compared with blood). Stimulation with MTB-specific antigens ESAT-6 and CFP-10 resulted in further increase, to 6.2 ng/mL (IQR: 0.0–11.0) and 3.6 ng/mL (IQR: 0.0–10.6) in the HIV-1-uninfected; and 2.7 ng/mL (IQR: 0.25–8.4) and 2.5 ng/mL (IQR: 0.25–8.3) in HIV-1-infected pericardial fluid respectively (*p*≤0.002 when compared with blood, Fig. 1). Although IFN-γ concentrations in the pericardial fluid of HIV-1-uninfected patients tended to be higher, a similar increase in response to antigen stimulation occurred in both HIV-1-infected and uninfected patient groups, with no statistically significant difference between them.

### Increased ESAT-6 specific IFN-γ-producing cell numbers in HIV-1-uninfected pericardial fluid

**Figure 2:**
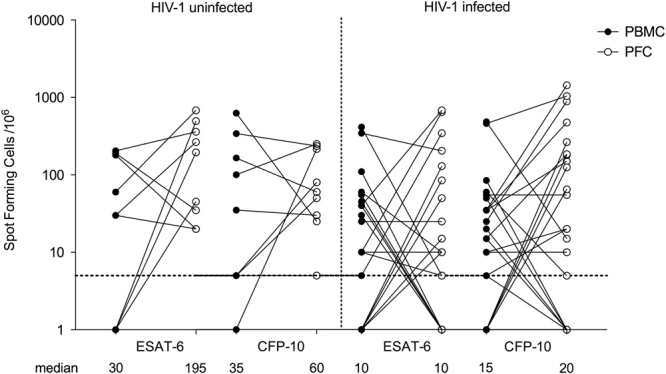
Increased numbers of ESAT-6-specific IFN-γ-producing cells in the pericardial fluid of HIV-1-uninfected patients. PBMCs and PFCs were assessed for antigen-specific IFN-γ responses by ELISpot assay. Each circle represents the number of IFN-γ spot forming cells/10^6^ cells in response to ESAT-6 and CFP-10, corrected for unstimulated control wells. The number of ESAT-6 specific IFN-γ spot forming cells/10^6^ cells was significantly higher in the pericardial fluid of the HIV-1-uninfected patients compared with HIV-1-infected patients (*p*=0.009, Mann–Whitney *U* test).

We hypothesized that the increased concentration of IFN-γ at the disease site would relate to increased numbers of antigen-secreting cells. Using the ELISpot assay with PBMCs and pericardial fluid cells (PFCs) of 9 HIV-1-uninfected and 23 HIV-1-infected patients, the frequency of IFN-γ-producing cells at the disease site compared with that in blood was determined (Fig. 2). ELISpot responses were detectable in both blood and pericardial fluid, but there was no clear evidence of increased numbers of antigen-specific cells at the disease site with significant heterogeneity between patients in response to all antigenic stimulants (Fig. 2 and Supporting Information Fig. 1). Comparing HIV-1 infected patients with uninfected controls, the only significant difference was the response to ESAT-6 in pericardial fluid, with a median of 10 SFC/10^6^ in HIV-1-infected patients compared with 195 SFC/10^6^ in HIV-1-uninfected patients (*p*=0.009).

### Increased numbers of CD4^+^ T cells at the disease site of HIV-1-uninfected patients

The trends towards lower numbers of antigen-specific T cells at the disease site in HIV-1-infected patients, together with the lower concentrations of secreted IFN-γ suggested that HIV-1 decreases the T-cell responses at the site of disease. To evaluate this further, we employed 4- and 8-colour flow cytometry to determine the surface phenotype and cytokine secretion of pericardial T cells. Supporting Information Figs. 2 and 3 illustrate the gating strategies.

Four-colour FACS on PBMCs and PFCs from 8 HIV-1-uninfected and 9 HIV-1-infected patients demonstrated an increased proportion of CD3^+^ lymphocytes in the pericardial fluid compared with blood in HIV-1 uninfected patients (median 86.9% IQR 72.6–90.5 versus 53.3% IQR 29.6–76.6, respectively, *p*=0.021, Table 2). In contrast, HIV-1-infected patients showed no significant difference between the proportions or numbers of CD3^+^ lymphocytes in blood and pericardial fluid.

**Table 2:** Characterization of lymphocytes from blood and pericardial fluid of pericardial TB patients, expressed as percentage (%) median (with IQR) and numbers per 10^6^ cells (with IQR)

	HIV-1 uninfected (*n*=8)			HIV-1 infected (*n*=9)			HIV-1 uninfected versus HIV-1 infected	
			
	Blood	Pericardial fluid	*p*-Valuea)	Blood	Pericardial fluid	*p*-Valueb)	*p*-Valuec)	
							Blood	Pericardial fluid
% Lymphocytes (FSC/SSC)	11.2 (6.5–31.6)	33.3 (18.2–48.1)	0.078	12.9 (11.1–22.6)	23.8 (18.4–29.9)	0.437	0.721	0.279
% CD3^+^ of Lymphocytes	53.3 (29.6–76.6)	86.9 (72.6–90.5)	0.021d)	58.8 (53.6–83.6)	91.2 (79.3–96.3)	0.094	0.382	0.065
Number CD3^+^ of Lymphocytes per 10^6^	51 133 (28 137–290 146)	198 138 (48 113–434 517)	0.148	147 107 (68 582–198 354)	185 074 (145 421–284 391)	0.094	0.345	0.950
% CD4^+^ of Lymphocytes	36.2 (14.3–44.8)	48.3 (16.9–62.0)	0.195	27.8 (12.2–40.7)	30.9 (19.6–50.5)	1.0	0.442	0.481
Number of CD4^+^ of Lymphocytes per 10^6^	35 281 (30 385–51 313)	140 562 (52 004–353 660)	0.023d)	26 598 (11 412–107 757)	36 190 (20 731–138 572)	0.297	0.613	0.121
% CD8^+^ of Lymphocytes	19.8 (14.5–41.1)	28.1 (20.0–44.2)	0.641	27.1 (17.9–47.8)	34.7 (22.5–55.2)	0.164	0.505	0.481
Number of CD8^+^ of Lymphocyte per 10^6^	1956 (1829–3352)	2950 (1224–18 104)	0.219	2486 (145 4801)	2721 (2019–6399)	0.461	0.694	0.867
% CD4^+^ HLA-DR^+^	15.1 (8–19.4)	14.9 (12.3–49.7)	0.195	6.7 (4.8–15.3)	15.9 (4.9–34.7)	0.148	0.083	0.798
% CD4^+^ CD71^+^	12.8 (8.5–16.1)	10.4 (4.3–20.1)	0.844	7.6 (3.0–11.9)	11.2 (7.2–32.0)	0.023d)	0.130	0.505
% CD^3−^CD56^+^CD16+ NK cells	14.1 (6.4–27.1)	1.7 (0.04–6.7)	0.035d)	6.2 (2.6–27)	6.4 (0.7–19.9)	0.312	0.673	0.297

a) Indicating differences between blood and pericardial fluid of the HIV-1-uninfected patients, using the Wilcoxon-matched pairs test.

b) Indicating differences between blood and pericardial fluid of the HIV-1 infected patients, using the Wilcoxon-matched pairs test.

c) Indicating differences between HIV-1-uninfected and HIV-1-infected patients, using the Mann–Whitney *U* test.

d) Gray shadow represents stastically significant difference.

Next, we assessed CD4^+^ and CD8^+^ T cells in the pericardium and found a shift from a predominantly CD4^+^ phenotype in the HIV-1-uninfected patients, towards a predominantly CD8^+^ phenotype in the HIV-1 infected. Thus, the median ratio of pericardial CD4^+^ to CD8^+^ T-cell proportions in HIV-1-uninfected patients was 1.71, and in the HIV-1-infected patients 0.66. This was reflected in significantly higher CD4^+^ T-cell numbers in the pericardial fluid compared with blood in HIV-1-uninfected patients (*p*=0.023, Table 2). These data support the previous finding that the predominant T cells are CD4^+^ in the pericardial effusion of HIV-1-uninfected patients, whereas CD8^+^ T cells are predominant in the pericardial effusions of HIV-1-infected patients 6.

Since HIV-1 infection causes generalized immune activation 29, we evaluated cell surface expression of HLA-DR and the transferrin receptor CD71 that represent activated and proliferating T cells 30. While there was no significant difference in the proportion of HLA-DR positive cells between blood and disease site in either patient group, proliferating CD4^+^CD71^+^ T cells were proportionally expanded (median: 11.2%; IQR: 7.2–32) in the pericardial fluid of the HIV-1-infected patients compared with blood (7.6%; IQR: 3–11.9; *p*=0.023, Table 2).

NK cells also produce IFN-γ in response to pathogens 31. We therefore hypothesized that CD3^−^CD16^+^CD56^+^ cells would be over-represented at the site of disease of HIV-infected patients. However, no significant difference between blood (median: 6.2%; IQR: 2.6–27) and disease site (6.4%; IQR: 0.7–19.9) was found; while in the HIV-1 uninfected patients NK cells were significantly lower at the site of disease compared with blood (median 1.7% IQR 0.04–6.7, and 14.1% IQR 6.4–27.1, respectively, *p*=0.035, Table 2). The greater proportion of NK cells in the pericardial fluid of the HIV-1-infected patients (although not significant) indicates that a proportion of IFN-γ produced at the site of disease originates from these cells.

### CD4^+^ memory T cells in the pericardial fluid of HIV-1-infected patients are less differentiated

**Figure 3:**
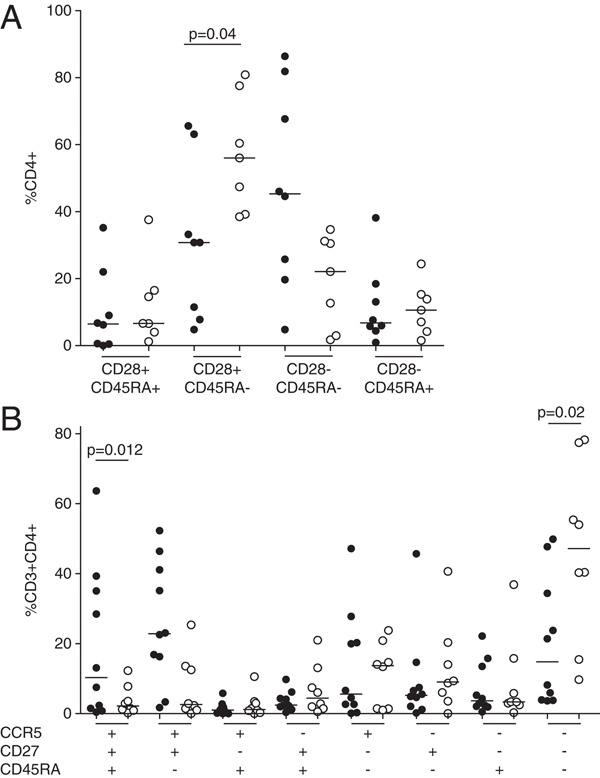
Flow cytometric analysis of CD4^+^ memory T cells in the pericardial fluid. (A) PFCs from HIV-1-uninfected (*n*=8, closed circles) and HIV-1-infected (*n*=7, open circles) patients were stimulated overnight in the presence of hkH37Rv, followed by surface staining for four-colour flow cytometry using CD4^+^, CD45RA and the co-stimulatory marker CD28. A significantly higher proportion of CD28^+^CD45RA^−^ cells at the disease site of the HIV-1-infected patients (*p*=0.04, Mann–Whitney *U* test), indicates a shift towards a less differentiated memory phenotype. Bars represent medians. (B) PFCs from HIV-1-uninfected (*n*=8, closed circles) and HIV-1-infected (*n*=10, open circles) patients were stimulated overnight in the presence of hkH37Rv, followed by surface staining for eight colour flow cytometry, including CD3, CD4, CD45RA, the co-stimulatory marker CD27 as well as CCR5 to phenotype the cells at the site of disease. The data indicate that CD4^+^ memory T cells in the pericardial fluid of HIV-1-infected patients lack the CCR5 receptor. A significantly higher proportion of CCR5^−^CD27^−^CD45RA^−^ cells (*p*=0.02), and significantly fewer CCR5^+^CD27^+^CD45RA^+^ cells (*p*=0.012) were found at the disease site of HIV-1-infected patients, compared with HIV-1-uninfected patients (Mann–Whitney *U* test). Bars represent medians.

We hypothesized that the memory phenotype of CD4^+^ T cells present at the disease site of HIV-1-infected patients would be different from that of uninfected patients, contributing to the differences we have described. First, we used 4-colour FACS with the combination of CD45RA and CD28 to characterize the memory phenotype of CD4^+^ T cells in pericardial fluid 9. We found significantly higher proportions of the less differentiated CD28^+^CD45RA^−^ CD4^+^ T cells in the pericardial fluid of HIV-1-infected patients (56.0%; IQR: 39.2–77.6) compared with HIV-1-uninfected patients (30.87%; IQR: 8.7–55.6; *p*=0.04, Fig. 3A).

Next, we employed multiparameter FACS for in-depth phenotypic analysis of CD4^+^ memory T cells in the pericardium of additional 10 HIV-1-uninfected and 9 HIV-1-infected patients (Fig. 3B). We found that the majority of CD4^+^ T cells at the disease site of HIV-1-infected patients did not display the disease site homing CCR5 receptor, with the dominant phenotype being CD45RA^−^CD27^−^CCR5^−^ (median: 47.2%; IQR: 21.7–71.2), significantly higher than in HIV-1-uninfected patients (median: 14.8%; IQR: 3.9–37.7; *p*=0.02). Similarly, terminally differentiated CD45RA^+^CD27^+^CCR5^+^ CD4^+^ T cells were infrequent in the pericardial fluid of HIV-1-infected patients (median of 2.2% IQR 1–6.7), compared with 10.3% (IQR: 1.3–36.1) in HIV-1-uninfected patients (*p*=0.012). Thus, our data suggest, that HIV-1-infection may eliminate the CCR5^+^ memory T cells, resulting in the recruitment of less differentiated memory T cells to the disease sites.

### Increased cytokine secreting capacity at the disease site irrespective of HIV-1 status

**Figure 4:**
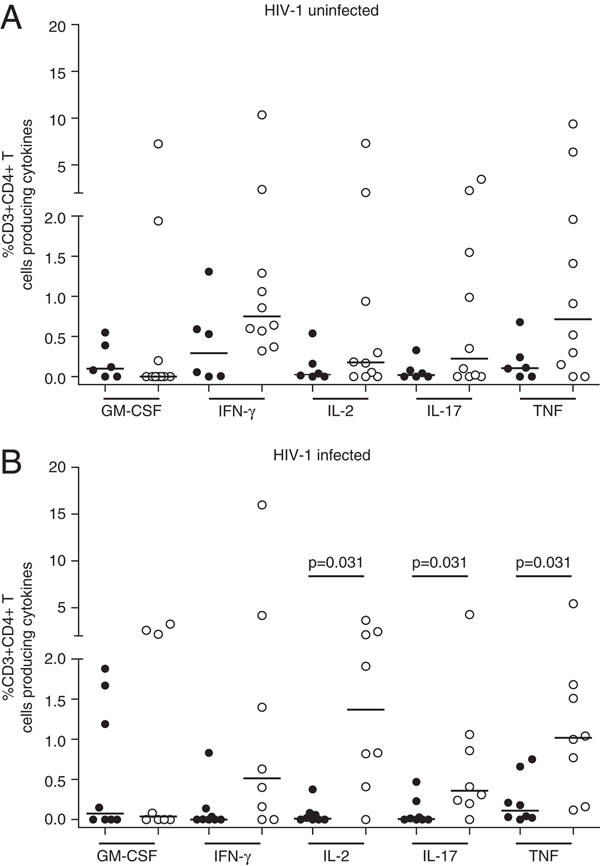
Increased cytokine-secreting capacity in the pericardium compared with that in blood, irrespective of HIV-1 status. Total cytokine secretion in response to ESAT-6+CFP-10 in blood (closed circles) and pericardial fluid (open circles) of (A) HIV-1-uninfected and (B) HIV-1-infected patients. Bars represent medians. Statistically significant differences between blood and fluid are indicated (Mann–Whitney *U* test).

Since the differentiation status of memory T cells affects their overall cytokine secreting capacity, we next investigated the CD4^+^ T cells in the pericardium that express IFN-γ, IL-2, TNF, IL-17 and GM-CSF, in response to antigen-specific stimulation (Fig. 4 and Supporting Information Fig. 4). There was a trend towards a higher proportion of cytokine expressing cells in the pericardial fluid compared with blood, significant in HIV-1-infected patients, with higher proportions of IL-2 (median 1.37% in fluid versus 0.01% in blood, *p*=0.03), TNF (1.02% in fluid versus 0.11% in blood, *p*=0.03), and IL-17^+^ cells (0.36% in fluid versus 0.01% in blood, *p*=0.03). These results are congruent with the presence of less differentiated cells at the disease site of the HIV-1-infected patients.

### Polyfunctionality of CD4^+^ T cells in pericardial fluid

**Figure 5:**
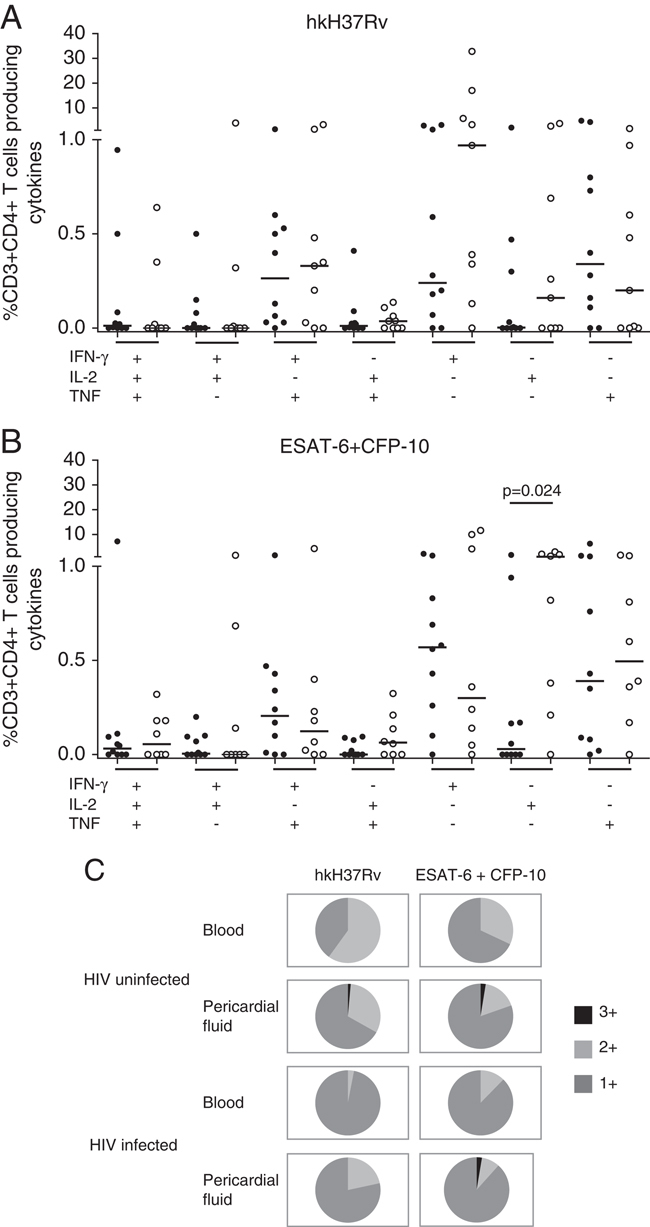
Polyfunctional CD4^+^ T cells are present at the disease site. CD3^+^CD4^+^ T cells were assessed for the expression of various combinations of IFN-γ, IL-2 and TNF in response to (A) hkH37Rv and (B) ESAT-6+CFP-10 stimulation, in the pericardial fluid of HIV-1-uninfected (closed circles) and HIV-1-infected (open circles) patients. (C) Pie-charts illustrate the overall proportional contribution of cytokine expressing cells to the antigen-specific response in blood and pericardial fluid. 1+: any one cytokine, 2+: any two cytokines, 3+: all three cytokines. IL-2 single-positive cells were proportionally expanded in the HIV-1-infected pericardial fluid compared with the uninfected in response to ESAT-6+CFP-10 (B, *p*=0.024, Mann–Whitney *U* test).

We next determined the presence of CD4^+^ T cells expressing combinations of IFN-γ, IL-2 and TNF, that have been described in the blood of TB patients and healthy controls vaccinated with novel TB vaccines 14, but have not been hitherto assessed at TB disease sites. We found no CD4^+^ T cells expressing all three cytokines in response to hkH37Rv or ESAT-6+CFP-10 in the blood, and very low proportions in the pericardial fluid of the HIV-1-uninfected patients (Fig. 5A and C). The majority of antigen-specific cells was single positive for IFN-γ or TNF, or expressed both IFN-γ and TNF.

In the HIV-1-infected group (Fig. 5B) there was more antigen-specific heterogeneity compared with the HIV-1-uninfected patients. Thus, in response to hkH37Rv, the majority of antigen-specific cells were single positive for IFN-γ, followed by cells double positive for IFN-γ and TNF. In response to ESAT-6+CFP-10 stimulation, the majority of cells were IL-2 single positive (*p*=0.024 compared with HIV-1 uninfected), followed by TNF single positive, and IFN-γ single positive. Interestingly, CD4^+^ T cells expressing all three cytokines were present in the pericardium only (Fig. 5C and Supporting Information Fig. 5). Thus, the pericardial fluid of HIV-1-infected patients showed a greater tendency towards polyfunctionality, consistent with the presence of less differentiated cells.

### HIV-1 viral load is increased in pericardial fluid

**Figure 6:**
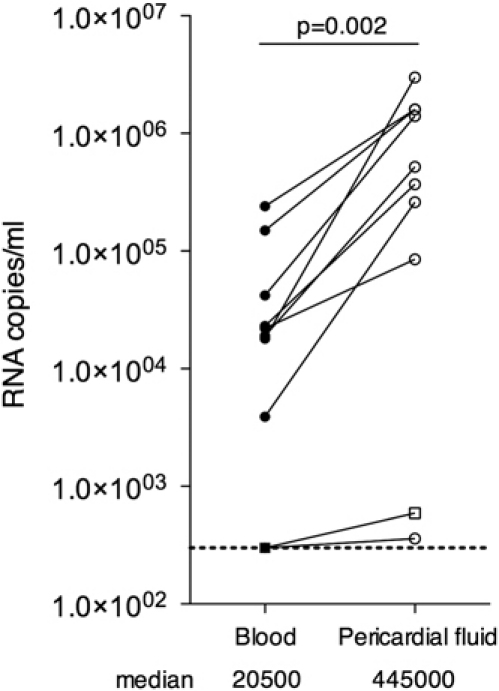
HIV viral load is increased in pericardial fluid compared with blood. HIV-1 RNA copies/mL were significantly elevated in matched pericardial fluid compared with serum from HIV-1-infected patients (*p*=0.002, Wilcoxon-matched pairs test). Squares represent the patient on ARV treatment at enrolment into the study.

It has previously been suggested that HIV-1 replication is increased at sites of MTB co-infection 32. To assess viral replication at the pericardial disease site, we determined HIV-1 viral load in paired serum and cell-free pericardial fluid from 10 HIV-1-infected patients. We found significantly increased HIV-1 viral load in the pericardium (median 445 000 copies/mL) compared with blood (median 20 500 copies/mL, *p*=0.002, Fig. 6), with an inverse correlation between the viral load and CD4^+^ T-cell proportions (Spearman *R*=−0.810, *p*=0.022) at the site of disease. A significant negative correlation between the memory cells most prominently found at the disease site of the HIV-1-uninfected patients (number of CD4^+^CD28^−^CD45RA^−^ cells/10^6^ PFCs) and pericardial viral load was also found (*R*=−0.76, *p*=0.05). These findings support that anatomically compartmentalized HIV-1 viral replication at extrapulmonary TB disease sites may be responsible for the skewed memory phenotype, possibly by local destruction of the CCR5^+^CD4^+^ T cells.

## Discussion

We have performed a detailed immunological investigation of cells from patients infected with two pathogens, MTB and HIV-1. By comparing blood and pericardial fluid from TB patients with and without HIV-1 infection, we show that the majority of CD4^+^ T cells at the disease site of HIV-1-uninfected patients express CCR5 and are of effector memory and terminally differentiated phenotype. HIV-1 infection results in altered MTB-specific memory CD4^+^ T cells at the disease site towards a less differentiated phenotype lacking the CCR5 receptor.

CCR5 is the chemokine co-receptor used by some strains of HIV-1 to gain entry into the CD4^+^ T cells. Using the simian immunodeficiency virus (SIV) model, macaques infected with the CCR5-tropic virus were unable to sustain the production of tissue homing CCR5^+^CD4^+^ memory T cells 33. Furthermore, it is known that HIV-1 preferentially infects and depletes CD4^+^ memory T cells, most of which are CCR5^+^, and this can affect lymphocyte trafficking to disease sites 34. Here, we show that the pericardium is a site of increased HIV-1 replication that could further contribute to localized destruction of CCR5^+^ cells. This may result in greater overall turnover of memory T cells, of a less differentiated phenotype. We speculate that in the absence of CCR5, MTB-specific CD4^+^ T cells use CXCR3 to migrate to the disease site. The lack of evaluation of CXCR3 is a limitation of this work, but the large amounts of CXCL10 detected in the pericardial fluid (data not shown) support this hypothesis.

We and others have previously demonstrated greatly elevated numbers of antigen-specific IFN-γ-producing T cells at the TB disease site using pleural fluid 11, 35, bronchoalveolar lavage fluid 36 and cerebrospinal fluid 37. Two case reports also describe the rapid diagnosis of TB pericarditis using the ELISpot assay 38, 39. Surprisingly, we found that the IFN-γ-specific ELISpot in pericardial fluid showed no clear concentration of antigen-specific IFN-γ-secreting cells, except for ESAT-6 in the case of HIV-1-uninfected patients. Compartmentalisation of ESAT-6-specific IFN-γ-secreting cells at the pericardial disease site exclusively in HIV-1-uninfected patients reflects the presence of more differentiated effector cells, congruent with our flow cytometric findings, and may indicate the contribution of these cells to host defense in containing MTB at sites of replication 11.

The ELISpot data were also surprising due to the apparent dissociation from the ELISA results that indicated increased IFN-γ secretion in pericardial fluid compared with blood, irrespective of HIV-1 status. This could be the result of the different detection methods, since cells in the pericardial fluid release IFN-γ and die, resulting in the accumulation of IFN-γ measured by ELISA. The ELISpot however, detects cells that produce IFN-γ and are present in the pericardial fluid at a given time (which is the time of pericardiocentesis in this case) and thus it does not account for the accumulated IFN-γ.

Intracellular cytokine staining experiments also demonstrated the potential of pericardial cells to secrete multiple cytokines compared with PBMCs in both HIV-1-infected and uninfected patients. Intracellular flow cytometry represents the effector potential of the cells rather than the actual effector function, since it stains cytokines intracellularly, only some of which would be eventually secreted (and thus be detected by the ELISpot assay). The fact that the difference was only significant in HIV-infected patients (for IL-2, TNF and IL-17), agrees with the less differentiated, more polyfunctional cytokine-secreting phenotype of the cells present at the disease site of these patients. Furthermore, increased IL-2 secretion was noted at the disease site of the HIV-1-infected patients only, and IL-2 secretion is characteristic of T cells with a less differentiated phenotype 9. These findings taken together with significantly elevated proportions of cycling (CD71^+^) cells at the disease site of the HIV-1-infected patients, support the presence of phenotypically less differentiated T cells.

Polyfunctional T cells that secrete multiple cytokines, most often studied as various combinations of IFN-γ, IL-2 and TNF, have recently been implicated as both a correlate of protection and pathology in TB, highlighting the difficulties of evaluating human immunity to TB 14. Our data show that low proportions of ESAT-6+CFP-10-specific triple-positive T cells were detectable at the disease site of both HIV-1-infected and uninfected patients. The presence of polyfunctional CD4^+^ T cells at the disease site of HIV-1-infected patients is consistent with the less mature phenotype of CD4^+^ memory T cells at the disease site. However, their presence at the disease site of the HIV-1-uninfected patients may support the recent suggestion that TB antigen-specific multifunctional T cells are better correlates of antigen load and disease status, than of protection 40.

Immune responses seen at extrapulmonary disease sites may be different from those seen in lung granulomas. A previous study comparing radiographically involved lung segment biopsies of pulmonary TB between HIV-1-infected and uninfected patients found higher proportions of lymphocytes in the involved segments of HIV-1-uninfected patients, together with lower proportions of CD4^+^ T cells in the HIV-1-infected patients 41. We similarly described significantly higher CD3^+^ lymphocyte proportions and CD4^+^ T-cell numbers at the disease site of HIV-1-uninfected patients.

It is recognised that HIV-1 viral load and HIV-1 replication can affect the maturation of CD4^+^ T cells responding to other pathogens, such as CMV, towards an earlier, less differentiated state which might adversely affect their ability to control those pathogens 12, 42. Our data indicate that the same appears true in case of MTB-specific CD4^+^ T cells. The significantly elevated viral load in the pericardial fluid compared with the blood further supports the idea, that HIV-1 replication at the disease site results in the presence of less differentiated cells, which may impair the ability of the immune system to control MTB replication. Early anti-retroviral treatment could have an important role in preserving the capacity of the immune system to control other pathogens.

## Materials and methods

### Patients

The University of Cape Town Faculty of Health Sciences Human Research Ethics Committee approved this study (reference number 402/2005), and all participants provided written informed consent. Patients with large pericardial effusions and suspected TB pericarditis were referred to the Cardiac Clinic at Groote Schuur Hospital, Cape Town, between February 2006 and December 2009. Each patient underwent echocardiography to assess the presence and size of pericardial effusion. When clinically indicated, pericardiocentesis was performed under echocardiographic guidance through a catheter. The case definition of pericardial TB has previously been reported 43, with either definite (positive for MTB complex in the pericardial fluid by culture or PCR) or probable TB pericarditis (empirical diagnosis by the clinical team, based on clinical signs, symptoms and response to TB treatment). Counseling and testing for HIV-1 were inclusion criteria for the study. Paired pericardial fluid and blood samples were collected from 74 patients in total (24 were HIV-1 uninfected and 50 were HIV-1 infected, Table 1). Due to limitation of cells available, not all assays were performed on all samples.

### Antigens

Antigenic stimuli were endotoxin-free and included the secreted Region of Difference-1 (RD1) encoded ESAT-6 (Rv3875) and CFP-10 (Rv3874) at a final concentration of 10 μg/mL. Additional antigens included α-crystallins Acr1 (Rv2031c) and Acr2 (Rv0251c), as well as the MTB cell wall-associated 38 kDa antigen (Rv0934, all at 10 μg/mL) for the IFN-γ ELISpot assay. The heat-killed (hk) laboratory strain of MTB H37Rv was used to stimulate cells for flow cytometry at a multiplicity of infection of 1 bacillus:1 cell (MOI=1:1), in order to achieve a more robust response. No antigenic stimulus was used as negative control, while positive controls included phytohemagglutinin (PHA, Sigma-Aldrich, 10 μg/mL), Purified Protein Derivative (PPD, SSI Denmark, 5 μg/mL) and staphylococcal enterotoxin B (SEB, Sigma-Aldrich, 10 μg/mL) according to the assay.

### Whole blood and pericardial fluid INF-γ restimulation assay

Undiluted pericardial fluid and whole blood obtained simultaneously were stimulated for 24 h with ESAT-6, CFP-10, PHA or no antigenic stimulus at 1 mL/well in 24-well tissue culture plates. Supernatant was removed and stored at −80°C until measurement of IFN-γ by ELISA as previously described 44.

### IFN-γ ELISpot assay

ELISpot analysis was performed as previously described 45, using cells from pericardial fluid and blood samples. PBMCs were separated using Ficoll, while cells from the pericardial fluid were isolated by centrifugation and washed with RPMI. Totally, 2×10^5^ cells in RPMI/10% FCS were plated per well of pre-coated IFN-γ MABTECH ELISpot plates. Antigens, including ESAT-6, CFP-10, Acr1, Acr2, 38 kDa, PPD were added at the doses described above, with PHA as the positive control and no antigen as the negative control, followed by incubation overnight (18 h) at 37°C with 5% CO_2_. The results are expressed as the number of antigen-specific spot forming cells per million (SFC/10^6^) corrected for the background (wells with no antigen).

### Flow cytometry and intracellular cytokine staining assay

PBMCs and PFCs from pericardial TB patients were stimulated for 18 h with hkH37Rv, followed by staining for surface markers including CD3-Allophycocyanin, CD4-Allophycocyanin, CD8-PerCP, CD45RA-PE and FITC, CD71-PE, CD28-FITC, CD16-FITC, CD56-PE and HLA-DR-FITC (all antibodies from BD Biosciences). A BD-FACS Calibur Flow Cytometer was used to acquire all cells. Isotype control antibodies and single stained samples were used to periodically check the settings and gates on the flow cytometer. Data analysis was performed using FlowJo Cytometry Analysis software (TreeStar, Stanford University, FlowJo Africa scheme).

Additional experiments for intracellular cytokine staining (ICS) combined with surface marker analysis, using an 8-colour panel were performed on a BD Biosciences LSRII flow cytometer. Following stimulation with recombinant ESAT-6 and CFP-10 (5 μg/mL each), hkH37Rv, PPD, SEB as the positive and no antigen as the negative controls, PBMCs and PFCs were surface stained for CD4-QDot 605 (Invitrogen), CD3-Pacific Blue, CD27-FITC, CCR5-PE, CD45RA-Allophycocyanin (BD Biosciences) and intracellularly for IFN-γ-AlexaFluor700 (eBioscience). Further cells were stained using CD4-QDot 605 (Invitrogen), CD3-Pacific Blue, IL-2-FITC (BD Biosciences), IFN-γ-AlexaFluor 700, IL-17-Alexa 647, GM-CSF-PE and TNF-PE Cy7 (eBioscience). Data analysis was performed with FlowJo Software (Tree Star, Version 8.6.6 from FlowJo Africa, http://www.flowjo.com/home/africa/) with Boolean gating to analyse cells with multiple fluorescent markers. Background subtraction was performed for all cytokine combination groups using the unstimulated negative control to exclude non-specific cytokine production. All negative values thus obtained were converted into zero for further analysis.

### Determination of HIV-1 viral load

Measurement of HIV-1 viral load was performed by the South African National Health Laboratory Services (NHLS) at Groote Schuur Hospital, using nucleic acid sequence-based amplification (NASBA) on stored serum and cell-free pericardial fluid samples from ten patients included in the FACS analysis experiments described above. The limit of detection by this assay was 300 copies of HIV-1 RNA/mL sample.

### Statistical analysis

Data were analysed using Graphpad Prism 5 Software (Graphpad Software). The normality of samples was tested using DŉAgostino and Pearson omnibus normality test. Normally distributed paired samples were compared by using a Studentŉs paired *t*-test, while non-parametric paired samples were analysed using the Wilcoxon-matched pairs test. Unpaired, non-parametric samples were tested using the Mann–Whitney *U* test and unpaired, normally distributed samples were compared using the unpaired Studentŉs *t*-test. A *p*-value <0.05 was considered statistically significant. Data are quoted as median (IQR) unless otherwise stated.

## Supporting information

Detailed facts of importance to specialist readers are published as ”Supporting Information”. Such documents are peer-reviewed, but not copy-edited or typeset. They are made available as submitted by the authors.
