# Supplementary material for: HIV-1 infection alters CD4+ memory T-cell phenotype at the site of disease in extrapulmonary tuberculosis
Source: Eur J Immunol. 2011 Oct 5;42(1):147–57. doi: 10.1002/eji.201141927 (PMC3298896; doi:10.1002/eji.201141927)
Supplement: Supplementary file 1 [file eji0042-0147-SD1.pdf]

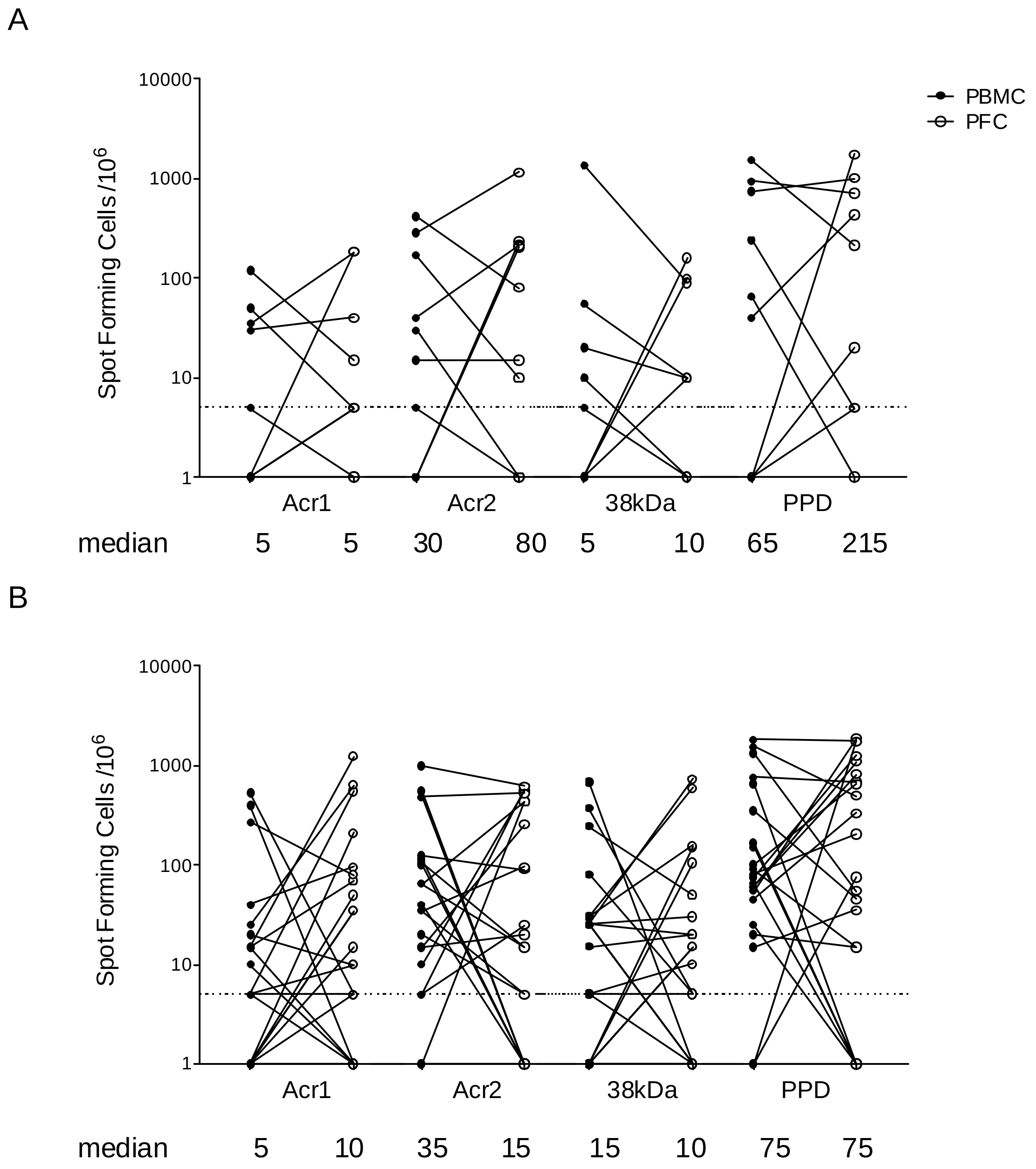

Supporting information, Figure 1

Heterogenous IFN-? ELISpot responses in blood (PBMC) and diseas site (PFC)

Each spot represents the number of IFN-? Spot Forming Cells / 10^6 cells in response to Acr1, Acr2, 38kDa and PPD in HIV-1 uninfected (A) and HIV-1 infected (B) pericardial TB patients.

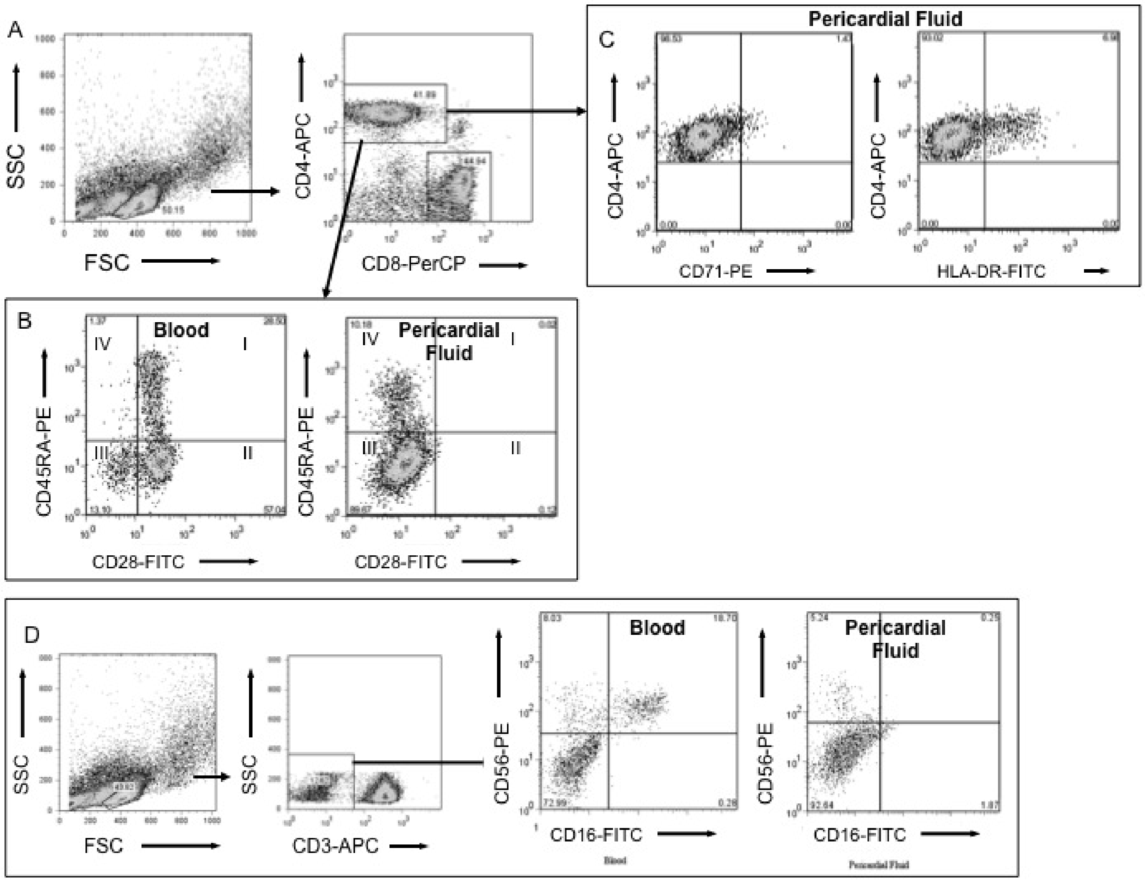

Supporting information, Figure 2

Representative scatter dot plots illustrating the gating strategy in four colour flow cytometry.

A. Selection of CD4 and CD8 positive cells: a scatter plot using Side Scatter (SSC) and Forward Scatter (FSC) was applied to select for lymphocytes. These were further divided into CD4 and CD8 positive cells by gating on CD4-PE and CD8-PerCP fluorochromes.
B. CD4 positive cells were further analysed for their surface expression of CD45RA-PE and CD28-FITC as follows (I) $CD4^+CD28^+CD45RA^+$, (II) $CD4^+CD28^+CD45RA^-$, (III) $CD4^+CD28^-CD45RA^-$, (IV) $CD4^+CD28^-CD45RA^+$ cells
C. CD4 positive cells were also analysed for their surface expression of CD71-PE and HLA-DR-FITC.
D. Representative example of gating on NK cells: CD3-APC negative cells were selected from the lymphocytes, and analysed as $CD16\text{-}FITC^+CD56\text{-}PE^+$ NK cells.

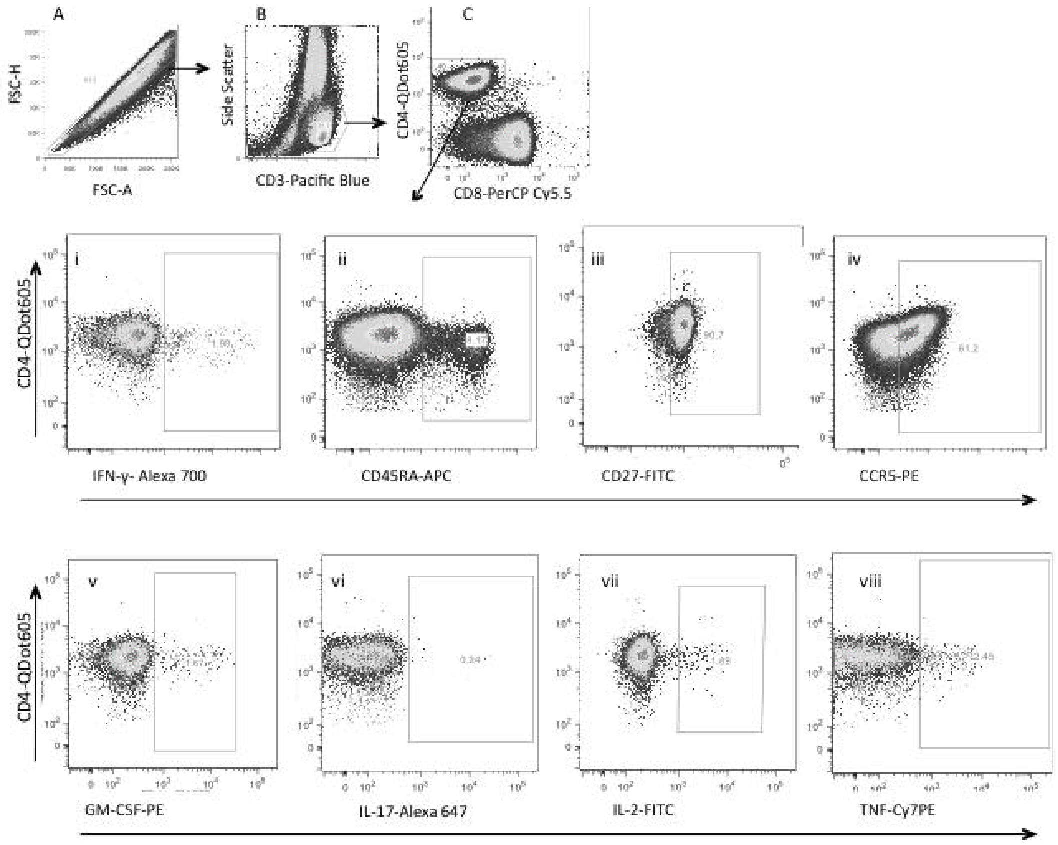

Supporting information, Figure 3

Representative scatter dot plots illustrating the gating strategy in eight colour flow cytometry.

Plotting FSC-Area against FSC-Height allowed to select for singlet cells (Panel A). $CD3^+$ cells were selected using SSC and CD3-PacBlue (Panel B), after which $CD4^+$ and $CD8^+$ cells were selected for by plotting CD4-Qdot 605 against CD8-PerCP Cy5.5 fluorochromes (Panel C). $CD4^+$ or $CD8^+$ cells on the *Y* axes were plotted against i. IFN-?-Alexa 700, ii. CD45RA-APC, iii. CD27-FITC, iv. CCR5-PE; and v. GM-CSF-PE, vi. IL-17-Alexa 647, vii. IL-2-FITC and viii. TNF-Cy7PE on the *X* axes, after which Boolean gating was applied to look at combinations of the markers.

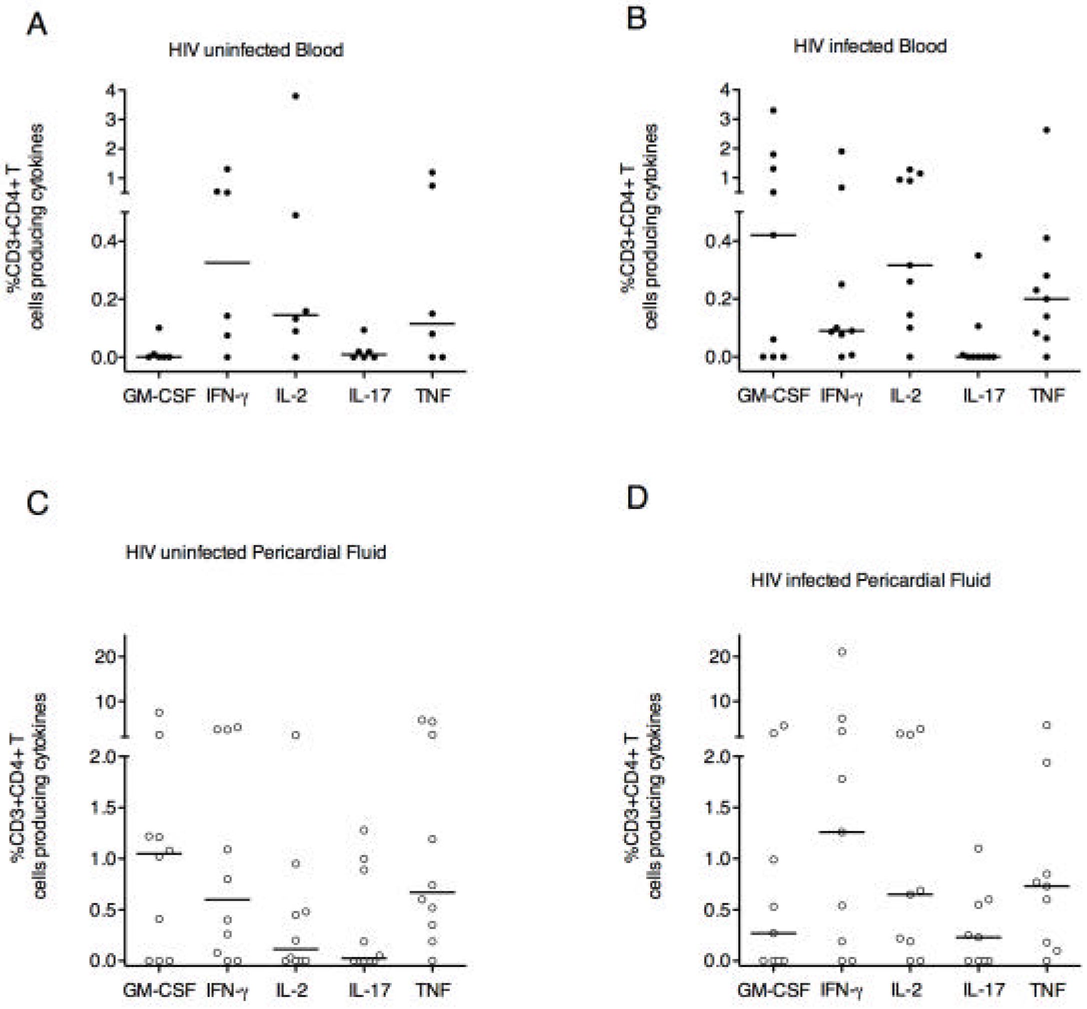

Supporting information, Figure 4

Total cytokine production in response to hkH37Rv in blood and pericardial fluid of HIV-1 infected and HIV-1 uninfected pericardial TB patients

Total cytokine production for GM-CSF, IFN-?, IL-2, IL-17 and TNF in blood (Panel A and B) and pericardial fluid (Panel C and D) for HIV-1 uninfected (Panel A and C) and HIV-1 infected (Panel B and D) pericardial TB patients.

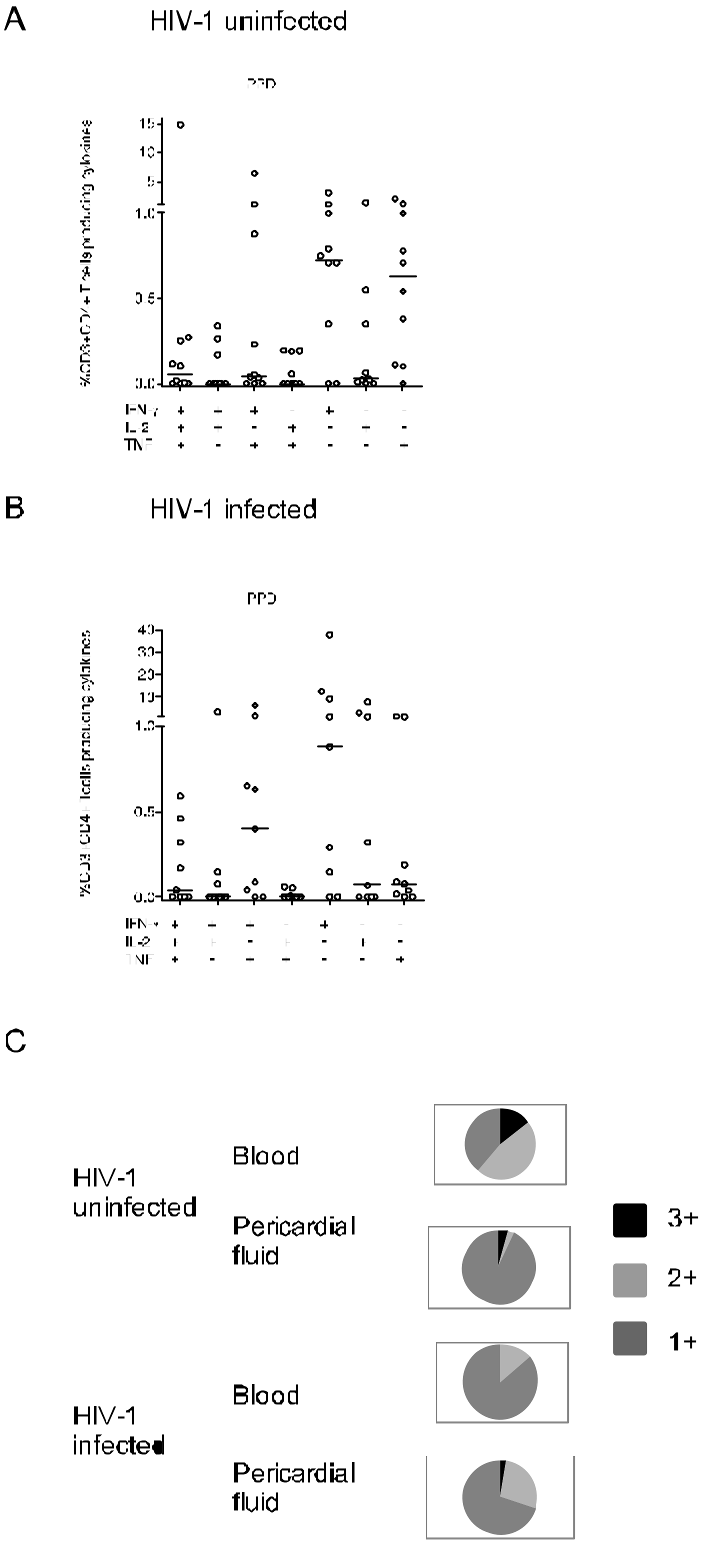

Supporting information, Figure 5

PPD specific polyfunctional CD3^{+}CD4^{+} T cells in the blood and pericardium

$CD3^+CD4^+$ T cells expressing various combinations of IFN-?, IL-2 and TNF in response to PPD stimulation, in the pericardial fluid of HIV-1 uninfected (panel A), and HIV-1 infected (panel B) patients. The pie-charts (Panel C) illustrate the overall proportional contribution of cytokine expressing cells to the PPD specific response in both blood and pericardial fluid. 1+: any one cytokine, 2+: any two cytokines, 3+: all three cytokines. IL-2 single positive cells were significantly increased in the blood of HIV-1 infected patients compared to HIV-1 uninfected patients (p=0.014).
